# Contextual Factors Explain Risk-Seeking Preferences in Rhesus Monkeys

**DOI:** 10.3389/fnins.2013.00007

**Published:** 2013-02-01

**Authors:** Sarah R. Heilbronner, Benjamin Y. Hayden

**Affiliations:** ^1^Department of Pharmacology and Physiology, University of Rochester Medical CenterRochester, NY, USA; ^2^Department of Brain and Cognitive Sciences, Center for Visual Science, University of RochesterRochester, NY, USA

**Keywords:** risk, gambling, neuroeconomics, uncertainty, macaque

## Abstract

In contrast to humans and most other animals, rhesus macaques strongly prefer risky rewards to safe ones with similar expected value. Why macaques prefer risk while other animals typically avoid it remains puzzling and challenges the idea that monkeys provide a model for human economic behavior. Here we argue that monkeys’ risk-seeking preferences are neither mysterious nor unique. Risk-seeking in macaques is possibly induced by specific elements of the tasks that have been used to measure their risk preferences. The most important of these elements are (1) very small stakes, (2) serially repeated gambles with short delays between trials, and (3) task parameters that are learned through experience, not described verbally. Together, we hypothesize that these features will readily induce risk-seeking in monkeys, humans, and rats. Thus, elements of task design that are often ignored when comparing studies of risk attitudes can easily overwhelm basal risk preferences. More broadly, these results highlight the fundamental importance of understanding the psychological basis of economic decisions in interpreting preference data and corresponding neural measures.

## Introduction

In 1996, Kacelnik and Bateson published a comprehensive review of the literature on animal risk preferences (Kacelnik and Bateson, 1996). They reported that, across 59 different studies, the majority of animals exhibited risk-averse preferences during gambles for food rewards. This pattern is generally concordant with the observation that humans are risk-averse for gains across a broad variety of contexts (Bernoulli, 1954/1738; Kahneman and Tversky, 1979). Here, risk is operationalized as the uncertainty in the possible outcomes of a decision, and can be mathematically specified as coefficient of variation (Weber et al., 2004). This usage is distinct from the everyday usage of the term, which is often synonymous with threat and necessarily involves the possibility of loss. The correspondence between human and animal data suggests that risk attitudes are evolutionarily ancient and are robustly stable across conditions (Chen et al., 2006). These results also tacitly endorse the validity of animal models for studies of risk preferences, and provide a foundation for neuroeconomic studies of risky choice (Platt and Glimcher, 1999; Fiorillo et al., 2003; McCoy and Platt, 2005).

In 2005, McCoy and Platt published the first study of the single-neuron correlates of risky choice (McCoy and Platt, 2005). In contrast to the large body of animal studies reviewed by Kacelnik and Bateson, they found reliable risk-seeking behavior in two rhesus monkeys (*Macaca mulatta*). Given a choice between a medium-sized squirt of cherry juice and a risky option that offered a 50% chance a large amount of juice and a 50% chance of a small amount, the monkeys reliably preferred the risky option, even though the expected values of the two options were matched. As the size of the large and small reward diverged, and risk level of the risky option thus increased, monkeys became even more risk-seeking. These monkeys even continued to prefer the risky option in a control experiment where the probability of winning was only 1/3 and the mathematical expected value of the gamble was lower than that of the safe option (McCoy and Platt, 2005). These risk-seeking preferences were not due to lack of training: the monkeys consistently chose to gamble even after months of experience with the task.

Since this early study, it has become clear that strong risk-seeking preferences are not unusual in macaques. The original monkeys continued to exhibit risk-seeking behavior for years, and six others from the same lab were consistently risk-seeking, totaling eight animals and hundreds of thousands of trials, with no cases of risk-aversion observed (Hayden and Platt, 2007; Hayden et al., 2008a,b, 2010; Long et al., 2009; Watson et al., 2009; Heilbronner et al., 2011). At least two other neurophysiology labs have also found reliable risk-seeking behavior in rhesus macaques (O’Neill and Schultz, 2010; So and Stuphorn, 2010). To our knowledge, no published study has reported stable risk-aversion in rhesus monkeys. With a variety of laboratories reporting the same result, one might conclude that risk-seekingness is some generalizable preference of rhesus macaques – perhaps distinguishing them from humans and other animals.

Here we argue the opposite: rhesus monkeys are not unique among animals, nor are they even inherently risk-seeking. Instead, we argue that, for practical reasons, the task design elements used by scientists who have studied risk attitudes in monkeys are those most likely to encourage risk-seeking. The most important of these elements are (1) decisions have very small stakes, a squirt or two of juice, (2) decisions are repeated hundreds or thousands of times with short delays (a few seconds) between trials, and (3) the reward structure of the task is learned through experience, rather than explained through language. These elements were preserved across studies in several laboratories in large part because they are optimal for neurophysiological recording, and they show up in rhesus macaque studies because monkeys are generally trained to gamble for the purpose of neuronal recording studies. Although the many studies listed above varied considerably from the original McCoy and Platt experiment, for example, by use of priming stimuli (Watson et al., 2009) and changes in cue presentation (Hayden et al., 2010), they still had the core features in common.

## Convex Utility Curves are Insufficient to Explain Risk-Seeking in Macaques

**Figure 1:**
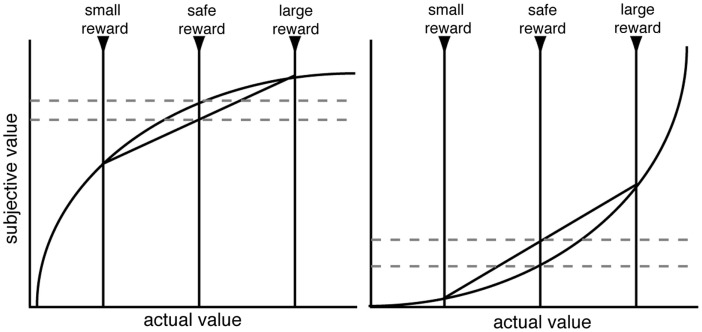
**Schematic of standard utility curve argument for risk-aversion (left) and risk-seeking (right)**. Decision-makers are assumed to have a non-linear shaped utility curve. When evaluating a gamble, decision-makers will utilize the computed expected utility rather than the expected value. With concave (or convex) utility curves, the expected utility is lower (higher) than the expected value. While utility functions are undoubtedly non-linear, it remains unclear whether the utility curve argument is sufficient to explain the range of risk preferences in macaques.

Before we address the factors that promote risk-seeking, it is helpful to discuss the most common explanation for risk-aversion or seeking: that risk-sensitive decision-makers have non-linear utility, and animals seek to maximize expected utility. Since the eighteenth century, it has been argued that decision-makers weight veridical reward values by a personal utility function, and these utilities, discounted by probability, are combined to form an expected utility (Bernoulli, 1954/1738). Thus, a concave utility curve, in which the marginal utility of each additional reward unit diminishes, is often assumed to explain human risk-aversion (Figure 1). These arguments are restricted to the domain of gains; for losses, hypothesized convex utility curves may explain risk-seekingness (Kahneman and Tversky, 1979). Indeed, in his commentary on the original macaque study (McCoy and Platt, 2005); Lee (2005) pointed out that the rhesus macaques’ behavior was consistent with a convex utility curve in the gains domain. However, while utility curves can *describe* risk preferences, it remains unclear whether they provide an accurate process model that *explains* risk preferences.

**Figure 2:**
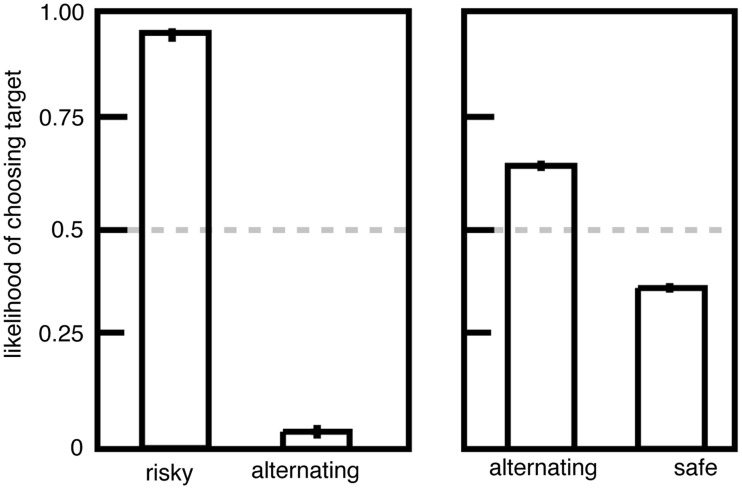
**Preferences for risky options over alternating ones are inconsistent with the standard utility curve argument**. Left: plot of preferences for risky (unpredictable) and alternating (predictable) options offering the same pairs of outcomes. Right: plot of preferences for alternating (predictable) and safe (fixed) options. Adapted from Hayden et al. (2008a).

We performed an experiment to investigate this question. We gave macaques a choice between two options, (1) a standard risky option with a 50/50 chance of a large and small juice reward and (2) an alternating option that provided either the large or small reward; its value alternated between these two reward sizes each time it was chosen (Hayden et al., 2008a). If non-linear utility functions drive risk-seeking preferences, then monkeys should be indifferent to risky and alternating options because the utilities must be the same. Instead we found that monkeys strongly and stably preferred risky options to alternating ones (Figure 2), indicating that the uncertainty itself biases the monkeys toward the risky option. In contrast, the alternating option was only weakly preferred to the safe option, suggesting that non-linearities in the utility function account for a small amount of risk attitudes.

Another utility-based account often used to explain risk-seeking, the energy budget rule, comes from foraging theory (Caraco et al., 1980; Caraco, 1981; Stephens and Krebs, 1986). The energy budget rule postulates that foraging animals should be risk-seeking if a large outcome means survival but the (ironically named) safe option means death. That is, if the minimum number of calories necessary to survive lies somewhere between the value of the safe option and the value of winning from the risky option, an animal should choose the risky option. It is theoretically possible that this preference, imbued by natural selection, is so strong that it affects monkeys in the laboratory, even though they are never even close to mortal danger. Although this is an appealing explanation for risk-seeking, is it highly unlikely to apply to gambling macaques. First, if the minimum number of calories necessary to survive lies somewhere between the value of losing from the risky option and the value of the safe option, the animal should always be risk-averse, not risk-seeking. Macaques are not limited to a single decision, but instead face hundreds or thousands of gambling choices every day. As the number of trials performed in a day increases, even when it is only into the double digits (and even more so beyond that), the optimal risk-sensitive foraging strategy rapidly comes to approximate risk neutrality. Consistent with this, satiety level (whether within a session or between sessions) does not affect risk preferences (Hayden, McCoy, and Platt, unpublished data). Thus, the criteria for the energy budget rule are quite strict, and, not surprisingly, there is inconsistent empirical evidence of risk-seeking behavior based on energy budget (Kacelnik and Bateson, 1997).

## Psychological Factors that Affect Risk Attitudes

Given the failure of traditional utility-based explanations, we next consider the possibility that specific contexts used in studying risk attitudes in monkeys influenced their preferences. Because these tasks were all originally developed for use in single unit physiology, studies of macaque gambling behavior are subject to unique constraints among the corpus of human and animal risk studies. They involve small stakes (to increase the number of trials performed in a session), a large number of trials (averaging neuronal responses over trials reduces the noise that comes from variability in neuronal firing patterns) presented very quickly (because neuronal isolation is unstable, physiologists collect data as quickly as possible), and task structures learned through experience (because monkeys have no language). We consider each of these factors here.

## Small Stakes

In humans, risk-aversion is weaker when the reward stakes are small (Holt and Laury, 2002; Fehr-Duda et al., 2010). Nobel-prize winning economist Markowitz, for example, intuited that typically risk-averse humans would prefer a lottery offering a 10% chance of $1 over a guaranteed 10 cents (Markowitz, 1952). Empirical tests of this bias, known as the “peanuts effect,” have demonstrated markedly reduced risk-aversion for small stakes (Hershey and Shoemaker, 1980; Green et al., 1999; Weber and Chapman, 2005). Some studies even suggest that small stakes may be sufficient to promote risk-seeking, although this is not fully demonstrated (Weber and Chapman, 2005). Consistent with this idea, some have argued that casinos effectively increase risk-seeking behavior among gamblers by dividing gambles into small (peanut-sized) amounts (Simmons and Novemsky, 2008). The reasons for the peanuts effect remains unclear, although it may reflect changing attitudes to disappointment for small amounts. Another possibility is that when reward sizes are small, gains loom larger than losses (Harinck et al., 2007). Indeed, there is some evidence that monkeys’ behavioral adjustments are more strongly motivated by the possibility of gains (or large rewards) than fear of losses (or small rewards) in their small-stakes gambling paradigms (Hayden and Platt, 2007; Hayden et al., 2008a), despite the well-known phenomenon of loss aversion for normal-sized amounts (Tversky and Kahneman, 1981).

Regardless of the psychological cause, it is clear that risk-aversion weakens greatly when stakes are very low. Primate gambling studies invariably use very small stakes – typically 0.1–0.3 ml of fluid per trial. The average daily intake for laboratory monkeys is generally around 2000 times greater than this amount. For the purpose of comparison, let us consider the human monetary equivalent. If an average American earns approximately $40,000 per year, or about $120 per day, the equivalent trial would offer 1/2000 of that amount, or 6 cents, lower than Markowitz’ “peanuts” amount. Although money and juice may not be directly comparable, it is safe to conclude that each individual trial offers such a small amount of juice that it probably puts monkeys into the domain of peanuts effects, and likely biases the monkeys away from risk-aversion and potentially toward risk-seeking.

Reward sizes in other animal studies run the gamut from small to large, and are of course difficult to equate to juice and money. For example, Abreu and Kacelnik (1999) gave starlings access to an average of 0.085 g of food crumbs on each trial, 0.4% of their average daily intake. This is four to eight times greater than the equivalent average monkey reward. In contrast, Kagel et al. (1986) gave rats approximately 0.2 ml of water per trial, which represents 4% of daily intake, an order of magnitude greater. Future studies using similar energy budget condition and trial structures should vary reward amounts and values directly, allowing for direct assessment of how much variability in risk preferences can be attributed to reward size. Nevertheless, Craft et al. (2011) demonstrated that rats are more risk-seeking when reward quality is low, matching the pattern from the human literature.

## Repeated Gambles

In a classic paper, Samuelson (1963) described a cafeteria meeting in which he offered his lunchtime companion a 50/50 gamble with two possible outcomes: winning $100 or losing $50. The possibly fictional colleague said he would reject the offer but would take it if it were repeated 100 times. Samuelson proceeded to mathematically prove the irrationality of this pair of preferences. Despite the attendant irrationality, the lure of serialization, which allows one to amortize one’s losses, is psychologically strong. Even upon being explained of its irrationality, many people persist in this set of preferences (Lopes, 1981). More generally, many researchers have found that serialization makes many types of gambles more attractive (Weaver, 1963; Lopes, 1981; Keren and Wagenaar, 1987; Wedell and Bockenholt, 1990; Hayden and Platt, 2009b). Preferences in repeated gambles often move toward risk neutrality (from risk-aversion for unique gambles), or even toward risk-seeking. In other cases, repeated gambles may elicit preferences that more closely match expected values (Keren and Wagenaar, 1987).

**Figure 3:**
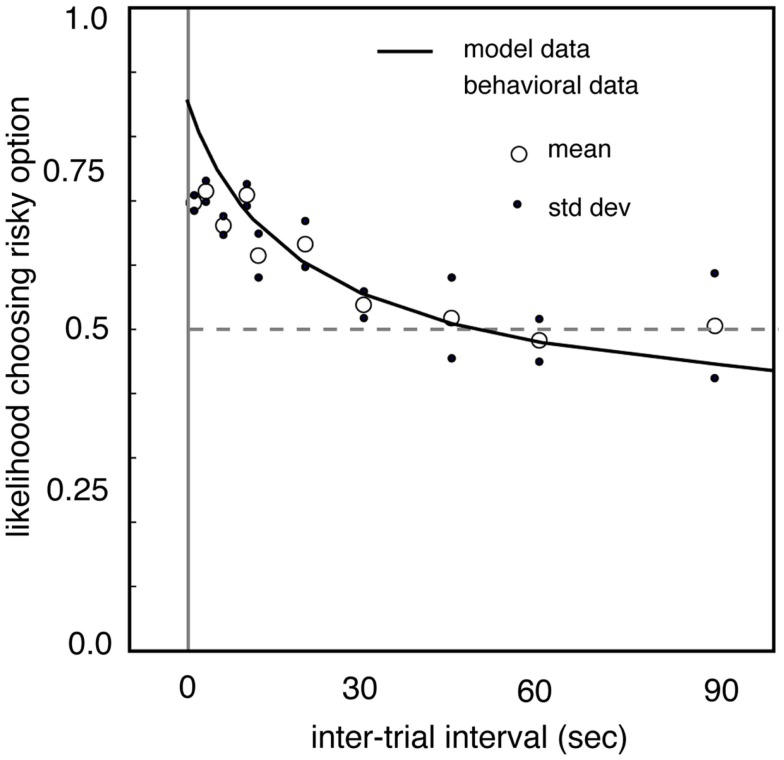
**Risk-seekingness of two macaques as a function of delay between trials**. Our observations indicate that inter-trial interval has a large effect on risk preferences. As delay between trials increases (horizontal axis), propensity to choose risky option (vertical axis) declines. When delay between trials reaches 90 s, monkeys are risk-neutral. These observations highlight the strong effect that rapid serial presentation of gambles has on promoting risk-seeking preferences. Adapted from (Hayden and Platt, 2007).

Interestingly, the frequency at which gambles are presented may impact preferences in a similar way, perhaps because frequency is good proxy for number of iterations. We tested this by measuring risk preferences in macaques in different blocks in which the delay between trials was controlled systematically (Hayden and Platt, 2007). When the inter-trial interval is lengthened from a few seconds to dozens of seconds, monkeys become significantly less risk-seeking (Figure 3). In our study, when the delay reached 90 s, the longest value tested, monkeys were risk-neutral. We speculate that delay affects the relative attention paid to the possibility of winning and losing, and that this change in attention affects preferences. Specifically, we developed a model in which monkeys estimated the expected time of the next large reward (and thus, in essence, differentially attended to winning) and then chose the option that would maximize the discounted value of the sequence leading to the large reward. (A control experiment confirmed that monkeys did not simply forget task details between trials). These results argue strongly for the importance of serial presentation of gambles on risk preferences, and more generally confirm the powerful importance of seemingly irrelevant details, like inter-trial interval, on gambling behaviors.

**Figure 4:**
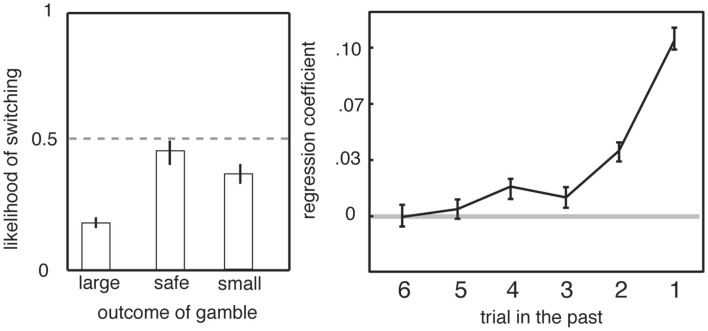
**Preferences in gambling task depend on recent outcomes**. Left: likelihood of switching from risky to safe strategy depends on outcome of previous trial. Right: preference depends on the outcomes of the five most recent gambles. Adapted from (Hayden et al., 2008b).

Another element of serial gambles that influences preferences is a strong bias toward adjusting behavior in response to recent outcomes (Barron and Erev, 2003; Hayden et al., 2008b). Gambling humans are susceptible to recency biases, including the hot-hand fallacy and the gambler’s fallacy. The hot-hand fallacy is the irrational belief that wins typically follow wins (Gilovich et al., 1985). Monkeys appear to be susceptible to the hot-hand fallacy, a pattern known in primate gambling studies as the win-stay lose-shift bias (Barraclough et al., 2004; Lau and Glimcher, 2005; Hayden et al., 2008b). That is, following a winning outcome, monkeys are more likely to choose the risky option again than if they have just experienced a loss (Figure 4). (The gambler’s fallacy, which has not been observed in monkeys, would have them predict that wins are “due” and thus more likely after a loss.) In addition, monkeys change their strategy based on surprisingness, meaning positive or negative deviation in outcome from expectation. Even though trials are independent, an unexpected outcome biases monkeys toward choosing the inferior option on the subsequent trial (Hayden et al., 2011). These changes in preference based on recent outcomes are large and robust; they can only be eliminated with extensive training (Lee et al., 2005). While this effect does not, by itself, explain risk-seeking, it demonstrates the importance of recent context, and not just present offer values, in governing preferences (see above and Haruvy et al., 2001; Hayden et al., 2008a).

As with reward amounts, ITIs and numbers of trials differ across animal studies. Even so, on average, the number of trials seems to be lower than in the monkey gambling studies, and the ITIs seem to be considerably longer. In the same starling study mentioned above (Abreu and Kacelnik, 1999), subjects did 36 trials per session, two sessions per day. The ITI was variable, but averaged 57.5 s. In the rat study (Kagel et al., 1986), the ITI was 146 s and subjects completed 17–41 trials per day. These numbers contrast drastically with monkeys’ hundreds to thousands of trials per day with ITIs of a few seconds. Clearly these are very different experimental environments, and may account for cross-species discrepancies.

## Feedback-Based Learning Promotes Risk-Seeking

In most studies measuring risk attitudes in humans, the subject learns about probabilities and rewards through written (or spoken) description rather than through experience. It is assumed that results from these studies have predictive validity to contexts in which gamble parameters are learned through experience. However, studies using repeated gambles in which contingencies must be learned from experience elicit strikingly different preference patterns in humans. Ido Erev and colleagues have shown that when humans rely on feedback instead of descriptions to learn about outcomes, they can become risk-neutral or even risk-seeking in the gains domain (and risk-averse for losses; Barron and Erev, 2003). The reasons for this discrepancy are currently unresolved (and reviewed in detail in Hertwig and Erev, 2009). One possibility is that subjects use overly small sample sizes in estimating probabilities; another is that they overweight low probabilities, as in prospect theory. Additional possible explanations include the recency bias observed in estimates based on memory, and biased mental sampling. Regardless of the ultimate cause, the fact that decisions from experience produce systematic biases is well-established (Hertwig et al., 2004; Hertwig and Erev, 2009).

Another factor that applies to experienced gambles, but not described ones, is information-seeking. In volatile environments, decision-makers will mix two simple strategies: exploitation (selecting the option thought to provide a greater expected value) and exploration (selecting the more uncertain – and thus more informative – option; Daw et al., 2006; Pearson et al., 2009). Indeed, in a dynamic foraging environment, monkeys and humans both have strong propensities to explore, routinely sacrificing rewards for the possibility to try new, more uncertain options (Daw et al., 2006; Pearson et al., 2009). In completely stable environments like those used in laboratory gambling studies in rhesus monkeys, exploratory sampling is theoretically unnecessary (and in fact costly); nevertheless, some baseline level of exploration may be innate, or perhaps the decision-maker assumes some inherent variability despite evidence to the contrary. Confirming the strong drive toward curiosity, monkeys will pay a premium to have uncertainty resolved earlier in the trial (Bromberg-Martin and Hikosaka, 2009). This drive for information may bias monkeys toward a risk-seeking strategy because choosing the risky option provides greater information about the range of possible outcomes in the environment than the safe one.

## Conclusions: Validity and Applicability to Other Contexts

We have identified three major features that may promote risk-seeking in rhesus monkeys: small stakes, repeated gambles, and learning from feedback. Preferences for risk are strongly dependent on these task parameters, and all published studies of risk attitudes in rhesus macaques have in common the three elements mentioned above. Future studies should directly manipulate these elements in isolation, thus testing the hypothesis that they are responsible for promoting macaque risk-seeking. This is not an exhaustive list of the psychological influences on risk-seeking; other factors that influence risk attitudes in monkeys include social milieu (Watson et al., 2009), background context (So and Stuphorn, 2010), and mood (Long et al., 2009).

The common features of task design in these studies suggest that risk-seeking preferences observed in macaques may not be an innate trait of their species. Indeed, recent studies optimized for physiology in rats have found risk-seeking preferences as well (Roitman and Roitman, 2010). In a similar vein, we have shown that when humans gamble in a paradigm designed to mimic, as closely as possible, that experienced by monkeys, risk-aversion disappears, and humans approach risk-seeking – and also show the same types of trial-to-trial hot-hand-like effects as monkeys do (Hayden and Platt, 2009a). In this study, we asked our human study participants to sit alone in an anechoic chamber with a juice tube placed in their mouth delivering squirts of juice in response to individual decisions in a task in which all rules were learned through experience. (Indeed, it was the same task used with monkeys, with the exception that humans used a keyboard rather than eye movements to signal their decisions). We speculate that if the study participants had been exposed to the same task for weeks and weeks, as the monkeys are, they may have become risk-seeking. These results are consistent with the observation that when humans and animals are placed in similar conditions, they exhibit similar gambling preferences (Weber et al., 2004). Thus, although there may be a main effect of species on risk attitudes (Heilbronner et al., 2008), it is likely to be overwhelmed by contextual factors when experimental conditions are not carefully standardized.

How do we reconcile these results with the pronounced risk-aversion observed in other animal species? Certainly, most animals are risk-averse (Kacelnik and Bateson, 1996), although there are now many known exceptions (for a review, see Heilbronner et al., 2009). As we have noted here, paradigms developed for neurophysiological recordings differ substantially even from most other animal choice studies. For example, ITIs are typically tens or hundreds of seconds rather than a few seconds, and animals may complete dozens of trials per day compared to hundreds or thousands. Handling times and reward values for seeds, pellets, and sucrose solution (Kacelnik and Bateson, 1996) may also differ drastically from those associated with drinking juice from a tube. Because most animal studies do find risk-aversion toward gains across a wide variety of methods, we should think of the conditions used in macaque gambling studies as somewhat extreme.

Broadly speaking, it is clear that attitudes toward risk are influenced by a large number of psychological factors, and that careful manipulation of these factors can push attitudes toward risk-aversion, risk-seeking, or neutrality. So far, the attributes of task design used to study risk preferences in rhesus monkeys bias them toward risk-seeking. These results highlight the importance of carefully considering the influence of task parameters when comparing across species, and if possible of using the same design elements. Of course, rhesus monkeys lack the ability to use language, and their conceptual representation of large numbers and explicit probabilities remains unclear. Given monkeys’ lack of language, it is quite possible that there may be no fair primate analog of standard written risk tasks in humans, just as there is no primate analog of jokes, irony, word-naming, or any other product of language. Thus, it may be impossible to use animals to model certain aspects of risky decisions in humans. However, it is also clear that humans and monkeys have similar behavior in response to gambles in which parameters are learned, suggesting that monkeys may be a good model for specific types of decision-making under uncertainty. Clearly, an important future goal will be dissociating the differences between preference patterns for different types of uncertainty (Volz and Gigerenzer, 2012).

Understanding the patterns of preferences for risk among rhesus macaques is critical to neuroeconomists – scientists who use measures of brain activity to infer the computational mechanisms of incentive-based (i.e., economic) decisions. Rhesus macaques are hoped to be a viable model for human economic preferences. Although a cursory examination of human vs. macaque preferences would suggest that they are quite different (in fact, opposite), here we have argued that similar psychological factors influence both species. Thus, macaque models of decision-making may accurately reflect the many cognitive biases influencing human risk preferences. These results therefore highlight the fundamental importance of identifying and accounting for the psychological processes behind decisions.

## Conflict of Interest Statement

The authors declare that the research was conducted in the absence of any commercial or financial relationships that could be construed as a potential conflict of interest.
